# ATP binding to neighbouring subunits and intersubunit allosteric coupling underlie proteasomal ATPase function

**DOI:** 10.1038/ncomms9520

**Published:** 2015-10-14

**Authors:** Young-Chan Kim, Aaron Snoberger, Jane Schupp, David M. Smith

**Affiliations:** 1 Department of Biochemistry, West Virginia University, 1 Medical Center Drive, Morgantown, West Virginia 26506, USA

## Abstract

The primary functions of the proteasome are driven by a highly allosteric ATPase complex. ATP binding to only two subunits in this hexameric complex triggers substrate binding, ATPase–20S association and 20S gate opening. However, it is unclear how ATP binding and hydrolysis spatially and temporally coordinates these allosteric effects to drive substrate translocation into the 20S. Here, we use FRET to show that the proteasomal ATPases from eukaryotes (RPTs) and archaea (PAN) bind ATP with high affinity at neighbouring subunits, which complements the well-established spiral-staircase topology of the 26S ATPases. We further show that two conserved arginine fingers in PAN located at the subunit interface work together as a single allosteric unit to mediate the allosteric effects of ATP binding, without altering the nucleotide-binding pattern. Rapid kinetics analysis also shows that ring resetting of a sequential hydrolysis mechanism can be explained by thermodynamic equilibrium binding of ATP. These data support a model whereby these two functionally distinct allosteric networks cooperate to translocate polypeptides into the 20S for degradation.

## Abstract (web summary)

The 26S proteasome contains a hexamer of ATPase subunits, which binds, unfolds and translocates substrates in an ATP-dependent manner. Kim *et al*. use FRET to show that ATP binding preferentially occurs at neighbouring subunits of the hexamer, and identify two allosteric systems that coordinate translocation.

## Graphical abstract

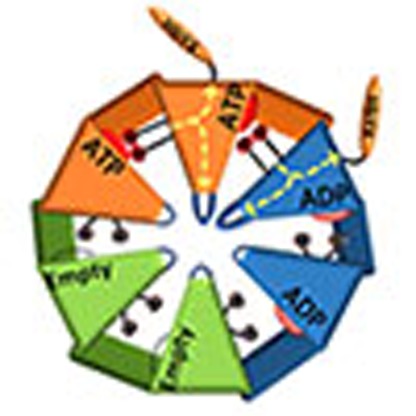

The 26S proteasome is an adenosine triphosphate (ATP)-dependent multisubunit protease complex that degrades polyubiqutinated proteins in a regulated manner. This 2.5-MDa compartmentalized protease contains ∼33 distinctive subunits in two major subcomplexes, the 20S core particle and 19S regulatory particle (RP/PA700). The 26S’s central regulatory hub is its hexameric ATPase complex (Rpt1-6), located at the 19S base. The ATPase ring’s N terminus (containing a coiled-coil domain) is intimately integrated with subunits involved in scaffolding, ubiquitin chain binding and processing. Its C-terminal side associates with the 20S proteasome via its C-terminal HbYX motif, which induces 20S gate opening to promote substrate entry. This architecture places the ATPase ring in a position where it can accept protein substrates on its N-terminal side, then, in an ATP-dependent manner, translocate them through its central pore and into the 20S for their degradation^1^,^2^,^3^,^4^. Archaea also have a proteasomal ATPase complex homologous to Rpt1-6 called PAN, which binds and similarly regulates the archaeal 20S proteasome.

Recent 26S cryo-electron microscopy (EM) analyses revealed that large conformational changes occur in the 19S when the ATPases bind ATPγS or substrates^5^,^6^,^7^. These similar ATP-bound and substrate-bound conformations are thought to be degradation competent. ATP (but not adenosine diphosphate (ADP)) binding to RPT1-6 or PAN triggers several essential steps required for protein degradation: (1) substrate binding^8^,^9^, (2) 19S–20S or PAN–20S association^10^,^11^ and (3) 20S substrate gate opening^10^,^11^. Therefore, ATP binding allosterically regulates conformational changes on both N- and C-terminal sides of the ATPase ring. Proteasomal ATPases unfold substrates when a substrate’s unstructured region binds the ATPases’ pore loops, which pull on the substrate upon ATP hydrolysis^12^,^13^,^14^. Substrate binding to these pore loops requires ATP to be bound, since empty or ADP-bound complexes do not bind substrates^8^,^9^,^15^. Conformational changes in the pore loops (due to ATP hydrolysis) translocate the peptide through the ATPase ring into the 20S, causing substrate unfolding^12^,^16^. The challenge is to understand how this complex network of subunit–subunit interactions allows ATP binding and hydrolysis to drive and coordinate the allosteric conformational changes that catalyse this process leading to substrate degradation.

ATP binding controls the ATPase ring’s C-terminal side by causing the HbYX motif to bind pockets between 20S α-subunits, allowing 19S–20S or PAN–20S complex formation and 20S gate opening^17^,^18^,^19^,^20^. The HbYX motif cannot associate with 20S α-subunits unless ATP binds to the ATPase. Although this mechanism is not completely understood, it is thought that ATP binding causes allosteric changes that allow association with the α-ring^17^,^18^. Averaged structures from 26S cryo-EM analyses (with ATP/ATPγS) showed densities for all three HbYX motifs bound to the 20S, confirming their necessity for 20S binding^5^,^21^,^22^. However, specific mechanistic details of ATP-dependent 26S assembly processes and gating functions are not clearly understood^10^,^11^,^15^,^17^,^19^,^20^,^23^,^24^. Understanding how ATP binding allosterically triggers ATPase–20S association via the HbYX motif will be key to understanding the dynamics of how these ATPase rings bind to the 20S to inject substrates.

Because ATP binding and hydrolysis to ADP are both essential for proteasome function, understanding how these events are coordinated both spatially and temporally is critical to understanding how work is done on substrates. Even though PAN has six identical subunits, it is known to have two high-affinity ATP-binding sites, two low-affinity sites and two sites that cannot bind ATP when high- and low-affinity sites are occupied^8^. ATP binding to the two high-affinity sites produces maximal function (for example, 20S gate opening or substrate binding), while ATP binding to the low-affinity sites reduces function. On the basis of similar functional studies, the 26S ATPases appear to bind ATP with nearly identical allosteries as PAN^8^. These and other data indicate the proteasomal ATPases’ highest functional state had two bound ATPs, two bound ADPs and two empty sites. Therefore, an extensive allosteric system linking the ATPase subunits must exist that controls how many ATPs bind around the ATPase ring. A working model for the ATP-binding/exchange reaction was built based on these data using symmetry considerations mirroring the nucleotide-binding pattern in the T7 helicase crystal structure^25^. This working model suggested that ATP binds to para-positioned subunits (180^o^ from one another) in the ring, but the results also did not exclude the possibility of other paired ATP-binding patterns^8^. However, strictly imposed allosteries indicated a patterned ATP-binding and hydrolysis mode is likely, which fits well with a sequential mechanism for ATP hydrolysis^26^.

A related AAA+ ATPase, ClpX, has similar high-, low- and no affinity ATP-binding subunits as the proteasomal ATPases, but any specific organizational pattern of ATP hydrolysis has not been established, though various models have been presented^27^,^28^. Interestingly, ClpX is capable of hydrolysing ATP (at impaired rates) with only a single fully functional (wild type (WT)) subunit. These results were interpreted to indicate that ClpX subunits have a degree of autonomy, which may allow ClpX to function in a probabilistic manner^29^. Because the ATP-binding and hydrolysis pattern is what coordinates and controls movements of the ATPase subunits, determining how these binding sites are organized spatially is essential to understanding how proteasomal ATPases function to translocate substrates. Determining if a specific ATP-binding pattern exists and what it might be is the first step to understanding how these molecular machines work.

AAA+ ATPases in all domains of life use six conserved motifs for function; that is, Walker A, Walker B, sensor 1, arginine finger (R-finger), sensor 2 and pore loops^30^,^31^,^32^. Out of these six motifs, only the R-finger is in a position to allow for allosteric communication between subunits^33^,^34^. The R-finger in proteasomal ATPases projects from one subunit into its neighbouring subunit’s nucleotide-binding site, and thus it functions in ‘*trans*’. In contrast, some AAA+ members, such as ClpX, do not appear to contain a functional *trans*-R-finger, but rather have a sensor-2 arginine functioning in ‘*cis*’^35^,^36^. Proteasomal ATPases have two conserved ‘trans’ arginines and either may function as an R-finger. Since a single arginine is sufficient to catalyse ATP hydrolysis in some members, it is not understood why some ATPases contain double arginines in this position.

Despite recent advances in determining the 26S proteasomes’ structure and dynamics, a detailed mechanistic understanding of how ATP binding and hydrolysis coordinate the conformational changes that generate a functional machine is not understood. In this study, we are interested in how ATP binding and hydrolysis allosterically regulate the proteasomal ATPases’ function. We found that PAN and the mammalian 26S ATPases bind ATP with an ordered neighbour-binding pattern (ortho) and that the conserved R-finger residues in PAN play a central allosteric role in controlling the fundamental mechanisms that catalyse proteasome function. In addition, these findings support an allosteric model, describing how ATP binding and hydrolysis are coordinated by separate allosteric systems that control the conformational changes that drive substrate unfolding and translocation into the proteasome for degradation.

## Results

### ATP binds to neighbouring subunits

The proteasomal ATPases have two high-affinity ATP-binding sites^8^. To determine the position of these high-affinity subunits within the hexameric complex, we monitored Förster resonance energy transfer (FRET) between fluorescent nucleotides bound to the high-affinity sites using a mant-ATP donor (m-ATP; Ex: 360, Em: 450) and TNP-ATP acceptor (t-ATP; Ex: 470, Em: 570; Förster critical distance (*R*_0_): 40 Å (ref. 37)) (Supplementary Fig. 1). To measure various distances between nucleotide-binding sites in the cryo-EM structure, we measured distances between Sensor 2 motif alanines (GAE/D) (PDB;4CR4). This residue was chosen because it binds adjacent to the nucleotide’s ribose ring, which contains the Mant or TNP moiety^38^. Supplementary Fig. 2 shows the estimated average distance between all pairs of ATP-binding sites in the 26S proteasomal ATPases.

To prevent ATP hydrolysis during the FRET experiment, we generated a PAN Walker B mutant (E271Q). ATP was shown to bind PAN-E271Q in a biphasic fashion exhibiting both high- and low-affinity binding sites, as was previously observed using WT-PAN and the non-hydrolysable ATP analogue ATPγS^8^ (Supplementary Fig. 3a). Because FRET efficiency will be determined in a mixed state (m-ATP+t-ATP), it is important to know whether their affinities differ to account for differential occupancy. ATP, m-ATP and t-ATP each bound to PAN-E271Q with remarkably similar affinities (Supplementary Fig. 3–c), indicating that fluorescent labelling of the nucleotide does not affect binding to PAN.

**Figure 1:**
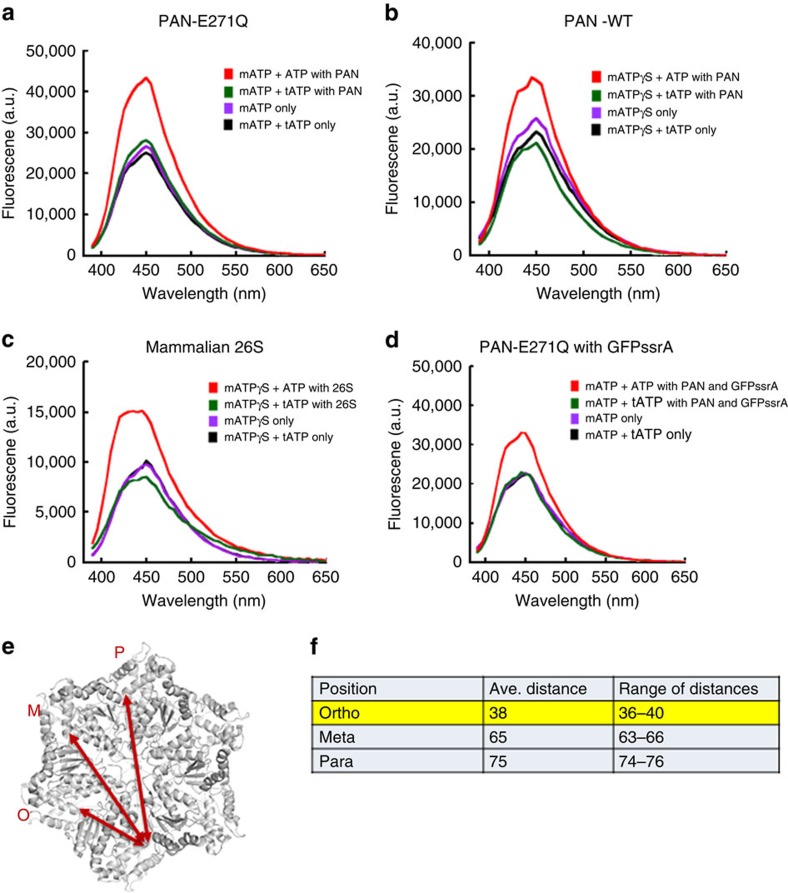
ATP binds to neighbouring subunits (‘ortho’ pattern) in the archaeal and mammalian proteasomal ATPases. (**a**) Emission spectra of m-ATP (1 μM) in the presence or absence of the indicated nucleotides (1 μM each) and PAN-E271Q (1 μM) at equilibrium (37 °C). The FRET (with t-ATP) and no-FRET conditions are shown and are colour labelled. (**b**) Same conditions as in **a** but with m-ATPγS and WT-PAN at 4 °C. (**c**) Same conditions as in **b** but with bovine 26S proteasome (1 μM) instead of PAN. (**d**) Same conditions as in **a**, but with the addition of GFP–ssrA (1 μM), which was photobleached by ultraviolet treatment before the assay to minimize the inner filter effect of GFP. (**e**) Structure of the 26S proteasomal ATPases (4CR4—atomic model derived from an 8-Å cryo-EM map), indicating the distance measurements between the sensor-2 residues in the various nucleotide-binding sites (*o*-ortho, *m*-meta and *p*-para). (**f**) Estimated average distance and ranges between ortho-, meta- and para-positioned nucleotide-binding sites in the eukaryotic 26S ATPases (4CR4) corresponding to **e**.

To determine FRET efficiency in the two-bound state, we monitored m-ATP (donor) fluorescence decrease in the presence of t-ATP (acceptor). To minimize unbound nucleotide while maximizing PAN population in the two-bound state, we used 2 μM of total nucleotide with 1 μM PAN hexamer (PAN^6^). This nucleotide concentration is saturating at ∼20 ×  *K*_d_ for high-affinity sites, and low-affinity sites will not be bound by any appreciable amount (*K*_d_=∼200 μM). PAN-E271Q was incubated with m-ATP and unlabelled ATP (no-FRET condition), or with m-ATP and t-ATP (FRET condition). In the FRET condition, m-ATP fluorescence decreases by ∼35% due to resonance energy transfer through TNP on t-ATP (Fig. 1a). To ensure the observed FRET was due to nucleotide occupancy of PAN-E271Q, m-ATP and t-ATP were mixed in the same conditions without PAN, and little FRET was observed (∼1%). FRET efficiency (see experimental procedures) was 0.67±0.07 (Table 1). Using the typical dipole orientation factor (*κ*^2^=2/3—randomly oriented) to calculate donor–acceptor distance, the observed distance between high-affinity binding sites is 37±2 Å. This is likely a good estimate since the fluorophores do not alter the nucleotide-binding affinity, and thus do not likely bind to PAN, allowing random rotation. This measurement indicates that high-affinity binding sites are ‘ortho’ (neighbouring) subunits. To eliminate assumptions about emission dipole orientation, we further measured anisotropy values of bound m-ATP and bound t-ATP and calculated a 0.14 *κ*^2^_min_ and 2.9 *κ*^2^_max_ (Table 1) yielding a 28–47 Å distance limit. Since the upper bound (47 Å) for this FRET pair is still 18 Å shorter than the meta position distance, these data further support the conclusion that high-affinity sites are neighbouring subunits (Fig. 1e,f). Since these FRET experiments are in ensemble conditions, the FRET distances are an average of all nucleotide-bound populations. Thus, if half of the population was meta and half was ortho the calculated FRET distance should be an average of these (that is, 52 Å). Because our calculated distance of 37 Å is very close to the average calculated ortho distance of 38 Å, we concluded that the vast majority of the bound population is ortho positioned. Moreover, because the obtained distances are consistent with minimum structural distances possible (ortho), the result is unambiguous as it could have been in the meta distance situation, which could be due to a mixture of ortho- and para-bound populations or a meta-only population.

Because these FRET measurements were made in a PAN Walker B mutant, we sought to verify ‘ortho’ binding in WT-PAN. To prevent ATP hydrolysis while using WT-PAN, we used m-ATPγS with t-ATP at 4 °C. This combination minimized t-ATP hydrolysis and allowed for stable fluorescence intensity measurements within a ∼60-s time frame. Identical experiments were performed under these new conditions using WT-PAN, and the distance determined by FRET was nearly identical to PAN-E271Q (Fig. 1b; Table 1). We also performed the same experiment with the 26S proteasome purified from bovine liver (Fig. 1c) and obtained nearly identical distance measurements as with PAN (Table 1), indicating the 26S proteasomal ATPases also have ‘ortho’-positioned high-affinity binding sites. This is the first evidence to show that high-affinity ATP-binding sites in archaeal and eukaryotic proteasomal ATPases are spatially organized in the same way. These data strongly suggest PAN and the 26S ATPases will bind and hydrolyse ATP in a similar fashion. This ‘ortho’ binding pattern is complementary to the helical topology of the 26S ATPases observed in several recent cryo-EM studies (see Discussion) and suggests PAN also shares this helical topology.

It is known that substrate (for example, green fluorescent protein (GFP)–ssrA) binding to PAN, which is ATP-binding dependent, stimulates its ATPase activity and that ubiquitin conjugates can act similarly on the 26S. Thus, to determine whether the ‘ortho’ binding pattern is altered in the substrate-bound state, we performed this same FRET experiment on PAN-E271Q with saturating amounts of photobleached GFP–ssrA bound to PAN. No change in donor–acceptor distance was observed compared with the same conditions without substrate (Fig. 1d; Table 1). Fluorescent GFP–ssrA binding to PAN-E271Q was confirmed by anisotropy. This result indicates the ‘ortho’ ATP-binding pattern is not altered when GFP–ssrA binds to PAN, and thus is not likely to be altered during substrate translocation. We next determined whether PAN’s R-fingers are required to generate the ‘ortho’ ATP-binding pattern, since these residues are known to play intersubunit allosteric roles in other AAA+ ATPases^30^,^33^.

### Selection of conserved arginines and generation of mutants

Sequence alignment of PAN and Rpt1-6 from human and yeast proteasomal ATPases shows two highly conserved arginines at residues 328 and 331 (Supplementary Fig. 4a). While the crystal structure of PAN’s nucleotidase domain is available, the subunit neighbouring contacts are not shown since it did not crystalize as a hexamer^38^. However, PAN’s structure was fit to 26S cryo-EM structures and analysis of the Rpt_2–1_ interface (PDB: 4BGR)^5^ clearly shows these two conserved arginines in Rpt2 projecting towards the Rpt1 nucleotide-binding site as expected for an R-finger (Supplementary Fig. 4b). Other Rpt interfaces show similar positions in each subunit, except the Rpt_1–5_ interface^5^. To systematically analyse the functional role of these two conserved arginine residues in PAN, we have generated three mutants: (1) R328A, (2) R331A and (3) R328/331A (double mutant), (Supplementary Fig. 4c).

### Both R-fingers required for ATP hydrolysis but not binding

**Figure 2:**
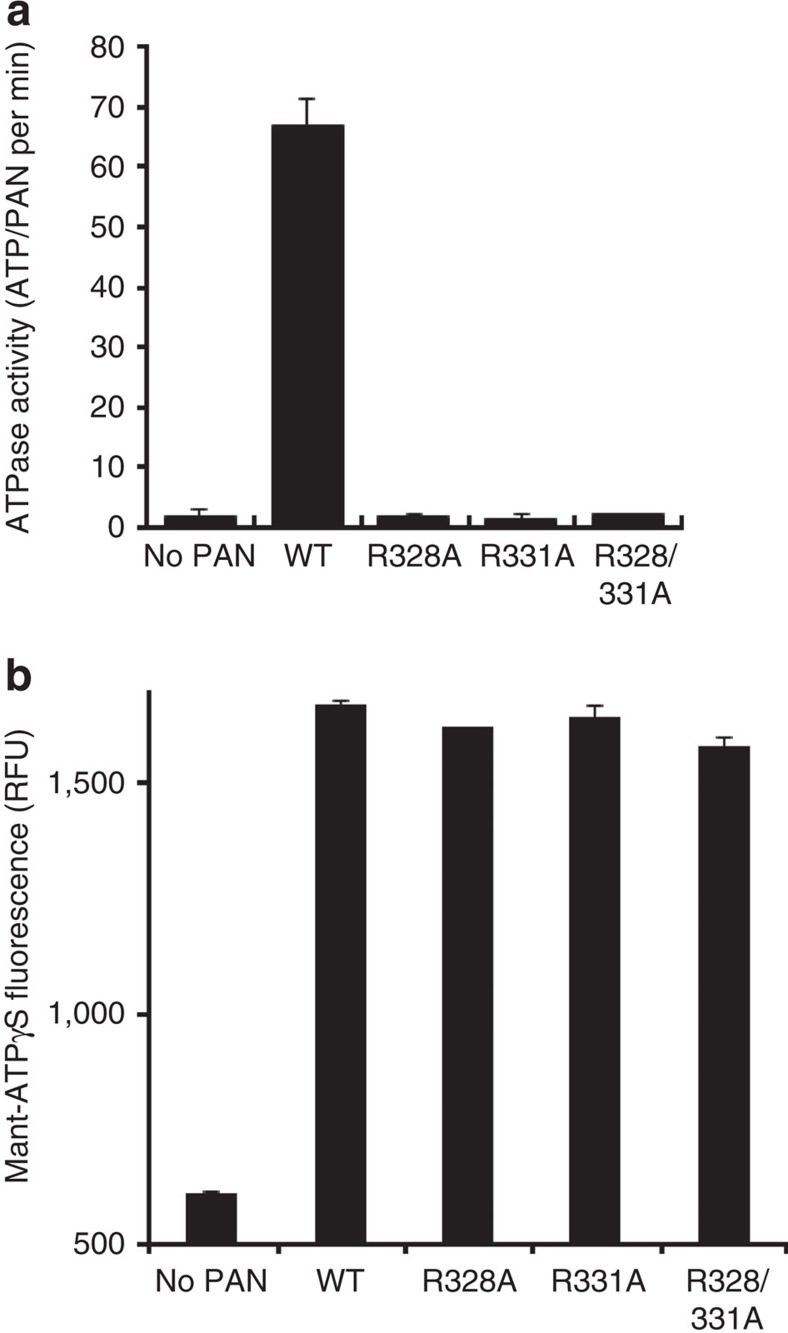
Both of the conserved arginines in PAN are required for ATP hydrolysis, but not for ATP binding. (**a**) Specific ATPase activity of WT and arginine mutants of PAN in the presence of 1 mM ATP. ATPase rates were determined with a real-time assay. (**b**) m-ATPγS (15 nM) binds to PAN and the arginine mutants (1 μM). Nucleotide binding is evident by a change in the intensity of mant fluorescence upon binding to PAN. Representative data are presented from three independent experiments ±s.d.

To determine whether these *trans*-positioned arginines are required to generate the ortho ATP-binding pattern, we first determined whether their mutation affected PAN’s ability to bind and hydrolyse ATP. The classical role of R-fingers is stabilizing the transition state’s negative charge on the gamma phosphate of ATP to catalyse its hydrolysis^33^. To determine whether R328 and R331 fulfilled this role in PAN, we used a real-time ATPase assay using an ATP-regenerating system. WT-PAN hydrolysed ATP at ∼1.1 s^−1^, consistent with prior observations using end-point assays^39^. However, no ATPase activity was found for any of the three arginine mutants (Fig. 2a). To study whether ATP could bind these mutants, we used the non-hydrolysable ATP analogue, mant-ATPγS (m-ATPγS). Mant–nucleotide fluorescence increases upon binding PAN^8^ and it activates the same functions as ATP binding (that is, PAN–20S association, 20S gate opening and substrate binding). m-ATPγS fluorescence intensity increased equally for WT-PAN and all three arginine mutants (approximately threefold) under enzyme-saturating conditions. Thus, while these conserved arginines are required for ATP hydrolysis, they are not necessary for ATP binding.

**Figure 3:**
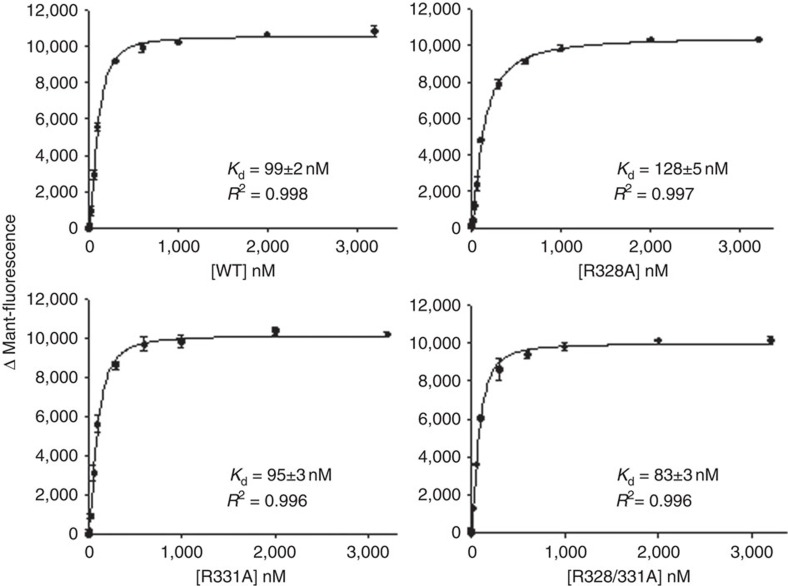
PAN’s conserved arginines are not involved in regulating ATP-binding affinity. Equilibrium ATP-binding affinity was determined by monitoring the change in fluorescence intensity of mant-ATPγS (15 nM) in the presence of increasing amounts of WT-PAN, PAN-R328A, PAN-R331A or PAN-R328/331A. The *x* axis is concentration of binding sites considering two high-affinity binding sites per PAN hexamer. The Michaelis–Menten binding hyperbola was fit to the raw data using nonlinear regression analysis to obtain the *K*_d_ (inset); the quality of fit (*R*^2^) is also shown.

Prior structural studies of the 26S ATPases indicated one of the six ATPase subunits have an R-finger not positioned in the active site^5^. Because of the important role these arginines may play in proteasomal ATPase structure and function, we next sought to determine whether binding affinities were altered. A mant–nucleotide saturation curve was generated by varying PAN concentration. m-ATPγS bound WT-PAN and all three arginine mutants with similar affinities (Figs 2b and 3). Calculated affinities are consistent with our prior ligand-binding study that quantified ^35^S-ATPγS binding to WT-PAN^8^. These affinities are also very similar to the m-ATP and t-ATP affinities for the PAN-E271Q (Supplementary Fig. 3b,c), indicating the γS modification does not affect affinity. However, because PAN is saturating in these experiments, we expect that only one nucleotide is bound to most PAN hexamers and thus, this method cannot reliably evaluate binding cooperativity.

### R-finger mutation does not disrupt ATP-binding stoichiometry

**Figure 4:**
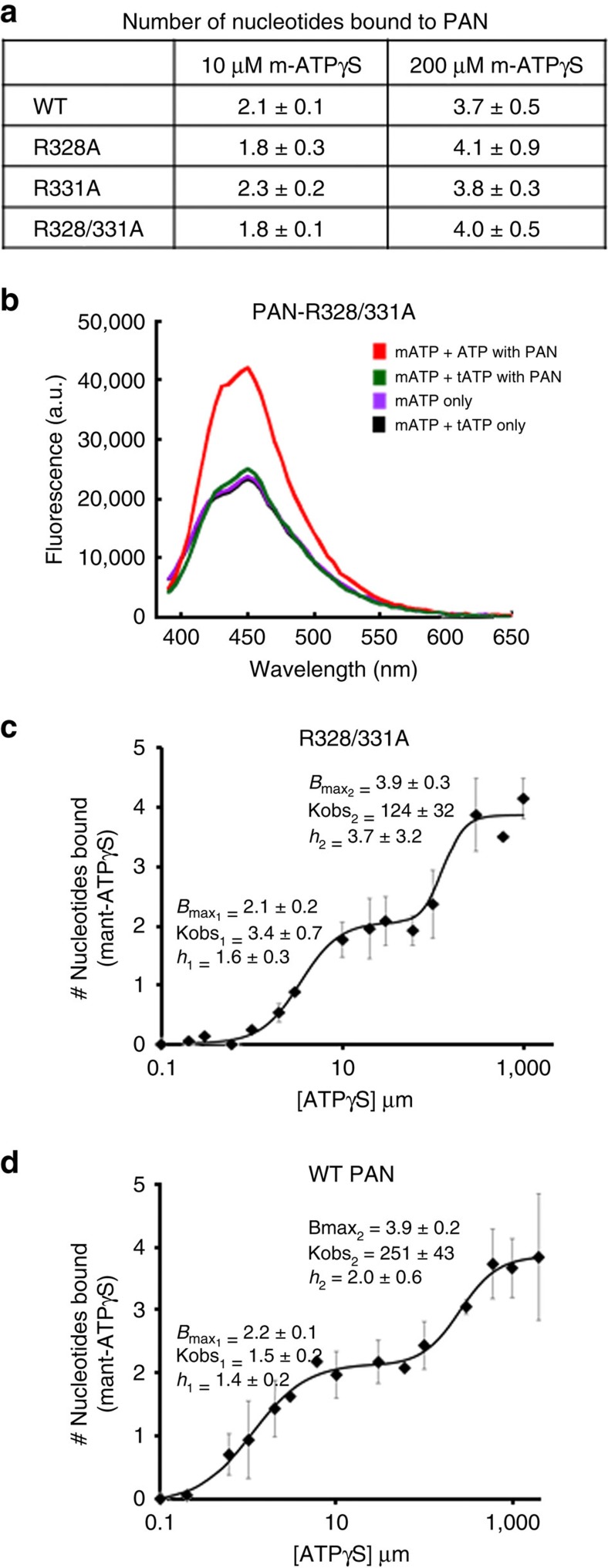
PAN’s conserved arginines are not involved in regulating nucleotide stoichiometry, or the ATP-binding pattern. (**a**) The number of m-ATPγS that bound to PAN (90 nM) was determined by rapid separation of bound nucleotide from free nucleotide using 100 μl spin columns at two different concentrations of ATPγS: 10 and 200 μM. Ten micromolar saturates only the two high-affinity sites, and 200 μM allows near saturation of the high- and low-affinity sites (ATP and ADP sites; ref. 8). The number of bound nucleotides per PAN hexamer was calculated for WT and each arginine mutant as labelled. Data are means of four independent experiments ±s.d. (**b**) Emission spectra of m-ATP as in Fig. 1a, but with PAN-R328/331A (1 μM). Quantifications are presented on Table 1. (**c**,**d**) The number of m-ATPγS-bound nucleotides to the labelled PAN variant was calculated as in **a** at increasing nucleotide concentrations to generate a binding curve. [PAN] was 200 nM and thus the free ligand bind approximation is not met here and thus the *K*-value is expressed as Kobs as it does not accurately quantify affinity. Representative data are presented from three independent experiments ±s.d.

PAN’s optimal functional state contains two bound ATPs^8^. At low ATP concentrations (10 μM), PAN binds two ATP molecules, but at higher concentrations (>60 μM), PAN will bind four ATPs (four-bound state), as long as hydrolysis is prevented by Walker B mutation (shown in Supplementary Fig. 3a) or by using a non-hydrolysable analogue. However, this four-bound state is strained and does not function optimally^8^. Thus, negative allosteries, which reduce affinity or completely prevent ATP binding, regulate ATP stoichiometry and binding pattern around the ring. To determine whether nucleotide-binding stoichiometry might be perturbed, we used rapid spin columns to quantify the amount of bound m-ATPγS per PAN hexamer for each variant. Two nucleotide concentrations were used: (1) 10 μM, which produces the two-bound state in WT-PAN, and (2) 200 μM, which produces the four-bound state. All arginine mutants bound the same number of nucleotides as WT-PAN (two- and four-bound states) (Fig. 4a). So, allosteries that regulate the nucleotide-binding pattern are not perturbed by R-finger mutation. To assess whether the ‘ortho’ ATP-binding pattern required conserved arginines, we performed FRET as in Fig. 1 using PAN-R328/331A (Fig. 4b; Table 1). FRET analysis showed that high-affinity ATP-binding sites were at the same distance from one another as WT-PAN (Table 1). This experiment also establishes that arginine mutation does not disrupt PAN’s quaternary structure. These combined ATP-binding analyses indicate neither R328 nor R331 in PAN plays any role in the allosteries that regulate ATP-binding affinity, ATP-binding cooperativity, ATP stoichiometry or ATP-binding pattern.

### ATP-binding cooperativity does not require the R-finger

To determine whether ATP-binding cooperativity is affected, we performed an equilibrium ligand-binding experiment with increasing m-ATPγS, using rapid spin columns to separate free from bound m-ATPγS. m-ATPγS bound PAN with two different affinities (low and high) showing a biphasic curve, as we have shown previously using ^35^S-ATPγS. The double-arginine mutant showed the same binding curve as WT-PAN (Fig. 4c,d), including a positive Hill slope for both curves. Furthermore, we observed positive cooperativity for WT-PAN (*h*=1.7±0.07) using a real-time ATPase assay (Supplementary Fig. 4d). This Hill coefficient slightly less than two is consistent with ATPγS-binding cooperativity for WT-PAN and arginine mutants, as well as m-ATP binding to PAN-E271Q. Importantly, the observed Hill coefficient (∼2) in all three experiments is also consistent with an average of two-bound nucleotides during operational conditions of ATP hydrolysis. These data demonstrate that neither R328 nor R331 is required for normal ATP-binding affinity, stoichiometry or intersubunit communication, which generates ATP-binding cooperativity.

### R-fingers are required for ATP-dependent substrate binding

**Figure 5:**
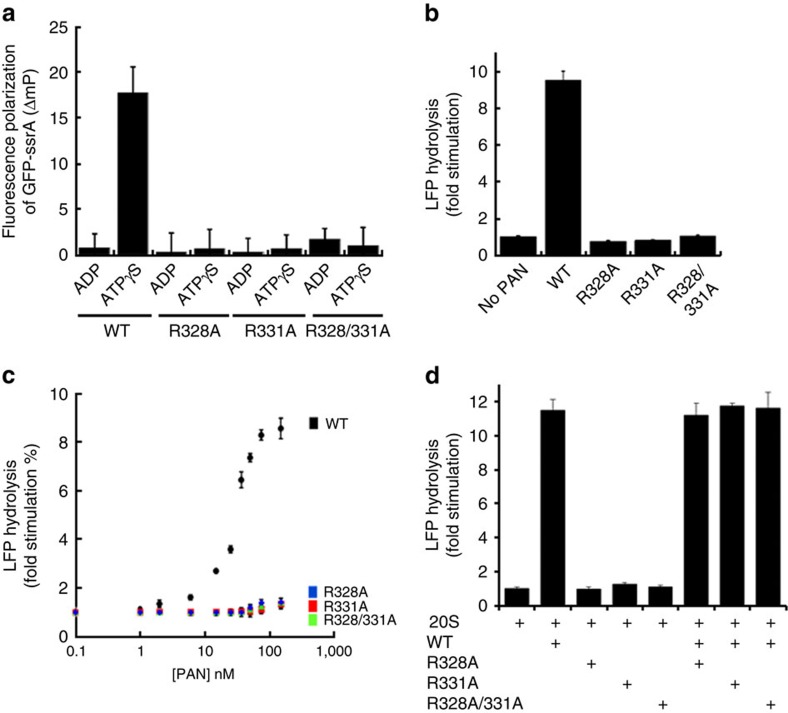
Mutation of either one of PAN’s conserved arginines abrogates ATP-dependent substrate binding and 20S gate opening. (**a**) Fluorescence polarization was used to monitor the binding of GFP–ssrA (0.08 μM) to PAN (0.12 μM) or its arginine mutants in the presence of 1 mM ADP (negative control) or 1 mM ATPγS. (**b**) Gate opening in the 20S proteasome (20 nM) by PAN WT, or its mutants (80 nM), was monitored with the LFP peptide hydrolysis in the presence of 10 μM ATPγS. ‘No PAN’ is 20S (archaeal) alone. (**c**) Gate opening in the 20S proteasome (20 nM) as a function of increasing concentration of WT-PAN and arginine mutants. (**d**) The gate-opening assay by the WT-PAN (10 nM) as in **b** but also in the presence of the other indicated PAN mutants (10 nM) to determine whether the mutants can compete with WT for binding to the 20S. All data are representative experiments and are the means of three independent measurements ±s.d.

Since ATP-binding affinity or binding pattern is not perturbed in the arginine mutants, we next asked whether the functional effects of ATP binding were altered. We first tested whether ATP-dependent substrate binding to PAN is affected by the mutation. We monitored GFP–ssrA binding to PAN using fluorescence polarization (FP). The unstructured ssrA tag allows binding to pore-1 loops in PAN and ClpX^13^,^40^. Binding ATPγS (but not ADP) to WT-PAN polarized GFP–ssrA by 17mP (Fig. 5a). This demonstrates that ATP binding to PAN is required to trigger GFP–ssrA association, which we have also shown previously^8^. However, none of the arginine mutants could polarize GFP–ssrA in the presence of ATPγS, indicating that both conserved arginines are necessary for ATP-dependent triggering of substrate binding. Therefore, these *trans*-arginines appear to detect the neighbouring subunit’s ATP-bound state and trigger allosteric conformational changes that allow substrate binding. For further verification, we tested PAN’s substrate-unfolding activity and substrate stimulation of PAN’s ATPase activity. The arginine mutants could not unfold GFP–ssrA (Supplementary Fig. 5a,b) and their ATPase activity was not stimulated by GFP–ssrA (Supplementary Fig. 5c), consistent with a loss of substrate-binding ability in the mutants.

### ATPase-20S association and gate opening require R-fingers

Besides substrate binding, another important ATP-binding effect is activation of PAN–20S association and gate opening^11^,^17^. ATPγS addition to WT-PAN and 20S stimulated LFP peptide hydrolysis approximately ninefold, demonstrating PAN–20S association and gate opening (Fig. 5b). In contrast, none of the mutants stimulated hydrolysis of the internally quenched nonapeptide, LFP. (Fig. 5b). To ensure arginine mutations did not simply alter PAN–20S affinity, we titrated PAN on the 20S to saturating concentrations. Only WT-PAN showed activation of the 20S (Fig. 5c). It is also plausible that PAN mutants bind the 20S without inducing gate opening; however, in a competition experiment none of the mutants could inhibit WT-PAN gate opening (Fig. 5d), indicating arginine mutants could not bind the 20S. These results indicate that although arginine mutants bind ATP with the same affinity and pattern as WT-PAN, they could not bind the 20S proteasome to induce gate opening. Therefore, both *trans*-arginines are required to allow allosteric communication of the ATP-bound state to the C-terminal HbYX motif to trigger PAN–20S association and 20S gate opening.

### R-fingers do not play a role in the ADP dissociation kinetics

**Figure 6:**
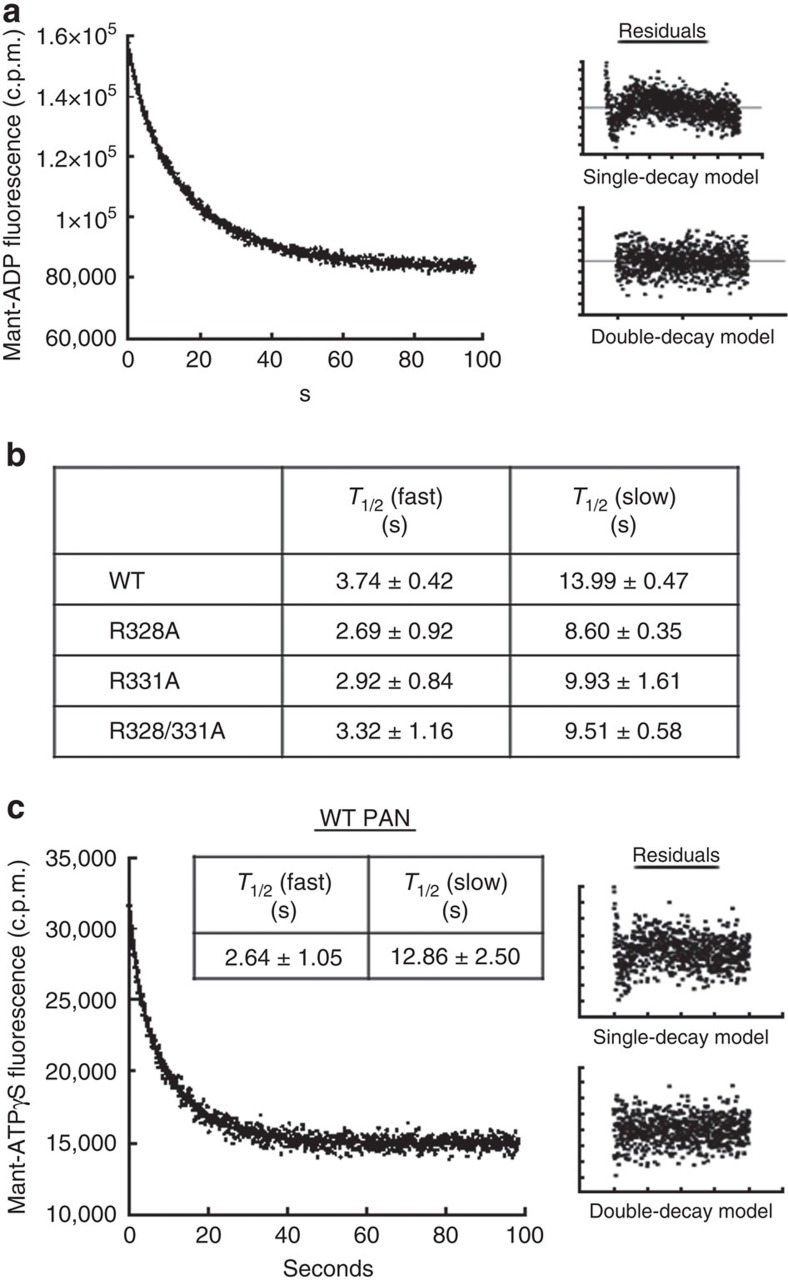
ATP and ADP off-rates are similar and the ADP off-rate is not affected by mutation of the arginine finger. (**a**) Pre-steady-state dissociation of the prebound m-ADP (150 nM) from WT-PAN (150 nM) was monitored by stopped-flow at 37 °C. Saturating amounts ADP (2 mM) were used to compete off the m-ADP. The residuals from fitting the raw data with single- or double-exponential decay models are shown (right). (**b**) The half-life (*T*_1/2_) of the bound m-ADP to WT-PAN and the arginine mutants for the double-decay model is presented, showing both fast and slow rates. (**c**) Pre-steady state dissociation of prebound m-ATPγS (1 μM) from WT-PAN (0.5 μM) was monitored as in **a**. Saturating amounts ADP (4 mM) were used to compete off the m-ATPγS. Residuals for the single- and double-decay models are shown (right). The determined half-life for both fast and slow rates for m-ATPγS are shown in the inset (double-decay model).

The role these two *trans*-arginines play in catalysis of ATP hydrolysis has not been investigated for proteasomal ATPases. At any individual active site, the ATP hydrolysis cycle goes through three primary steps: (1) ATP binding, (2) transition state formation and gamma phosphate cleavage (and release) and (3) ADP dissociation (required to allow ATP binding in the next round of catalysis). ADP dissociation is thought to be rate-limiting in this process. Since we already know step one, ATP binding, is not affected by arginine mutation (Figs 2 and 3; Table 1), either step 2 or 3 must be perturbed to cause the observed loss of ATPase activity. To determine which step is impaired, we performed a stopped-flow experiment to determine whether the ADP off-rate was affected by arginine mutation. PAN (150 nM) was preloaded with equimolar m-ADP (150 nM) in the first injection syringe. These concentrations allowed for ∼80% of the m-ADP to bind. The second syringe contained the same buffer with saturating ADP (2 mM), and the two samples were injected together pneumatically. Mant fluorescence was monitored every 100 ms during the competition experiment, and raw data were fit to single- or double-exponential decay curves. A single-exponential decay curve did not fit any of the generated curves, but a double-exponential curve fit well with appropriate residuals (Fig. 6a). These fits suggest that PAN contains two types of sites for bound ADP, each with a slightly different off-rate. Since PAN is equimolar with m-ADP, it is expected that most PAN has a single m-ADP bound while some contained two bound m-ADPs; the presence of these two different bound populations could explain why two different decay rates are observed, but other explanations are possible. Conserved arginine mutation has little to no effect on the ADP dissociation rate, especially for the fast step (Fig. 6b). In fact, mutation of both arginines actually increased the fast and slow off-rate by 11 and 32%, respectively, and the fast-rate change was not statistically significant. Therefore, the complete ATPase activity loss caused by conserved arginine mutation is expected to be due to a loss of ability to stabilize the transition state that catalyses γ-phosphate cleavage (that is, step 2 is defective), rather than due to a decreased ADP off-rate.

### ATP on/off is in equilibrium between hydrolysis events

To probe the process of ATP hydrolysis around the proteasome’s ATPase ring, some groups individually deactivated some but not all of the subunits in an ATPase ring^15^,^41^,^42^. Interestingly, a mutation of only a single subunit (1 of 6) has only a minor impact on ATP hydrolysis rates when the substrate is not bound^15^,^41^. This suggests that either: (1) some subunits in the WT ring never bind and hydrolyse ATP (that is, there is no subunit switching and thus the inactivation of inactive subunits does not impact hydrolysis rates), or (2) all subunits can hydrolyse ATP, but deactivated subunits can be skipped or partially ignored. The former explanation was convincingly ruled out, at least in ClpX studies, where subunit conformational switching occurred^28^,^43^. In addition, the existence of multiple ring conformations in the 26S ATPases also suggests subunit conformational switching in the proteasomal ATPases to some extent. The later explanation (#2) suggests that strictly sequential mechanisms of ATP hydrolysis are not possible, and the Sauer group proposed that a probabilistic model can explain this subunit-skipping phenomenon^29^,^43^. In the probabilistic model, any subunit (with a bound ATP) can bind and hydrolyse ATP, but some subunits can have a higher probability of binding or firing presumably depending on which subunits are currently bound to ATP. However, our data indicate that ATP binding is highly ordered and controlled, at least in the proteasomal ATPases, suggesting a strictly sequential ATP hydrolysis mechanism. But how can a sequential mechanism be rectified with subunit skipping? To answer this question, we sought to determine whether ATP can leave PAN without being hydrolysed; if so, ATP could bind new subunits, resetting their position in the ring. If this occurs on a timescale relevant to ATP hydrolysis rates, this could explain how subunit skipping occurs in a mechanistically sequentially functioning ATPase with minimal impact on ATP hydrolysis rates. To determine this, we performed a stopped-flow experiment similar to Fig. 6a, but this time prebound m-ATPγS to WT-PAN in one syringe, and competed with saturating ADP (4 mM) in the other syringe. ADP was used as a competitor rather than ATPγS, because the competitor will bind to the low-affinity sites (ADP sites) first, which has no observable effect on PAN function^17^, before competing at high-affinity sites. Alternatively, if ATPγS was used as a competitor, it would induce a strained four-bound ATP state, which may alter normal off-rates. Again a single-exponential fit did not produce satisfactory residuals but a double-exponential decay fit well (Fig. 6c). The fast off-rate was 2.6 s, which was slightly faster but comparable to WT-PAN’s ADP off-rate. The slow off-rate was 13 s, also similar to the slow ADP off-rate. Thus, ATP has a similar off-rate as ADP. Since the ADP off-rate is rate limiting (that is, new ATPs cannot bind and be hydrolysed until ADP leaves), approximately half the ATP that binds PAN leaves without being hydrolysed. This indicates that ATP binding is in thermodynamic equilibrium (coming and going) during the ATP hydrolysis cycle to such an extent that ring resetting likely occurs between ATP hydrolysis events during normal operation. The impact of thermodynamic ring resetting is also expected to be greater on an enzyme exhibiting the dwell/burst kinetics proposed for ClpX^44^, due to lengthy pauses in the cycle. Therefore, these results demonstrate that thermodynamic ring resetting can explain how subunit skipping can occur in a strictly sequential ATPase, especially when ATP hydrolysis is perturbed (or delayed) in single-subunit mutants.

## Discussion

**Figure 7:**
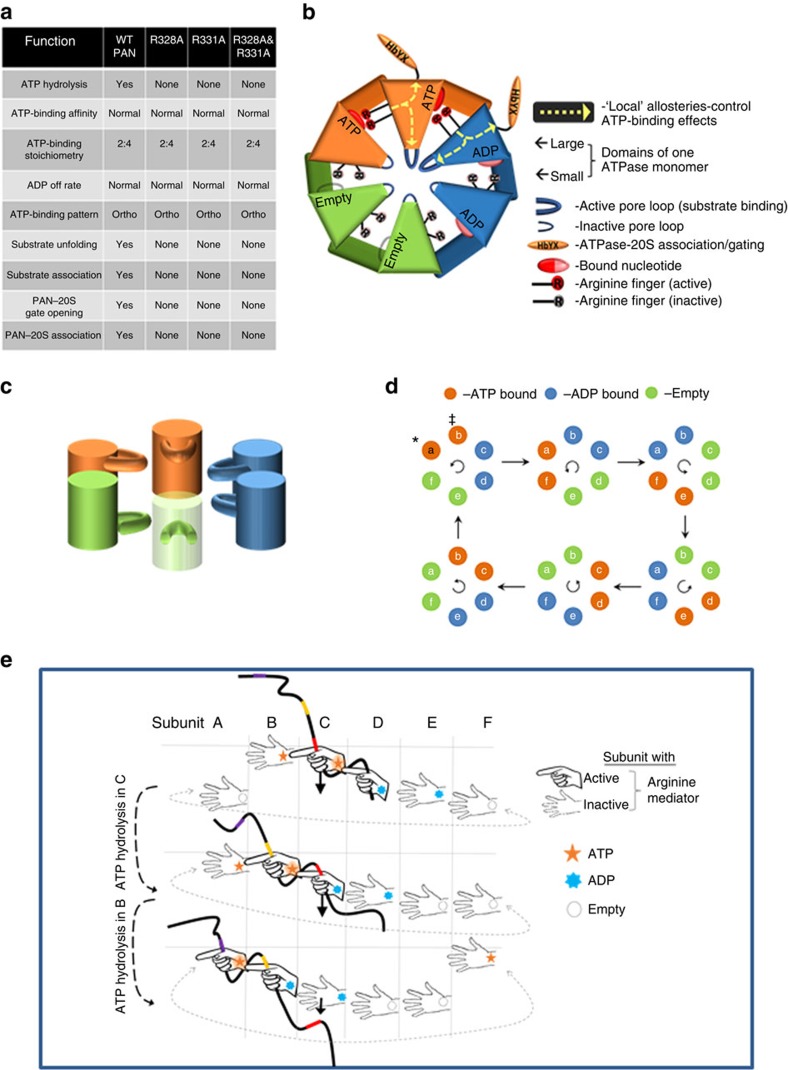
Allosteric nucleotide-binding/exchange and substrate translocation models for the proteasomal ATPases. (**a**) Summary of the roles that conserved arginines play in mediating ATP-binding effects in the proteasomal ATPase PAN. (**b**) Ortho ATP-binding model for the proteasomal ATPase PAN, and the local allosteries that are mediated by the conserved R-finger residues (yellow arrows), which control the depicted ATP-binding effects. The *trans*-functioning R-finger (requiring both conserved arginines) contacts ATP in its counterclockwise neighbour (Walker subunit), this allosterically triggers substrate binding and HbYX exposure in its own subunit (arginine subunit). The ortho-nucleotide-binding pattern is controlled by the global conformation of the ATPase ring, in coordination with the helical topology is not controlled by the local allosteries of the R-finger. (**c**) A model depicting nucleotide-bound lockwasher-like topology of 26S ATPases after substrate engagement. Colour coding of this helical topology would correspond to the colour coding in **b** to depict rotation of the helical conformation with subunit progression. (**d**) Model of ATP hydrolysis subunit progression undergoing one complete cycle. Individual subunits a–f remain fixed as ATP is hydrolysed with a single-subunit progression around the ring starting with the lagging ATP-bound subunit. The nucleotide bound to each subunit is indicated in the key by colour. The leading (*) and lagging (‡) subunits are indicated in the first iteration (top left). The top-left and bottom-right configurations in this cycle could correspond to the two resting states of the 26S ATPases observed in cryo-EM studies mentioned in the discussion (with either Rpt1 or Rpt3 at the top positions). (**e**) Model depicting protein translocation in proteasomal ATPases. The ortho ATP-binding pattern and the local allosteries of the *trans*-R-finger are combined to demonstrate how ATP binding and hydrolysis could result in translocation of engaged substrate. Here the six subunits of the ATPase ring are peeled open and the height of the pore loop (hand) is indicated by vertical position. The closed hand with finger indicates a subunit whose R-finger is contacting its neighbour’s bound ATP, and thus has affinity for substrate. Therefore, ATP hydrolysis in this subunit results in movement of the substrate downwards without losing its ‘grip’ on the substrate, at which time the next subunit can also bind after ATP binds to its neighbour. In this way the substrate is never released until its translocation is completed, thus generating a mechanism for highly efficient unidirectional translocation into the 20S proteasome.

Here we have shown PAN has two conserved arginines that are both required for ATP hydrolysis, confirming their expected, but unverified, role as an R-finger. But why do some AAA ATPases have two arginines in this position rather than just one, when one arginine is sufficient for catalysis in many different oligomeric nucleotidases^30^,^34^? One possibility is that this dual-R-finger arrangement is more efficient at sterically transmitting allosteric changes—due to ATP binding—between neighbouring subunits. Here we found that the proteasome requires this dual R-finger to trigger two specific ATP-binding-dependent functions: (1) for triggering substrate binding and (2) for inducing HbYX-dependent PAN–20S association coupled to 20S gate opening (Fig. 7a). This dual R-finger in the proteasomal ATPase PAN thus performs two independent functions: (1) catalysing ATP hydrolysis and (2) allosterically regulating mechanistically critical conformational changes.

Because the R-finger in the proteasomal ATPases structurally functions ‘*in trans*’ (PDB:4BGR), the allosteric effects are carried out by the subunit containing the R-finger (arginine subunit) and not the one bound to ATP (Walker subunit), since ATP can bind the Walker A/B motif but cannot trigger ATP-binding effects without both arginines present (Fig. 7b). One explanation is that the OB domain promotes hexamerization (all mutants run as a hexamer by native gel), while the arginine mutation disrupts ATPase domain interaction leading to their separation. However, this possibility is ruled out since the ATP-binding sites are positioned at exactly the same distance in WT and the double mutant (Fig. 4b), implying the mutant’s quaternary structure is intact. Therefore, ATP binding in the Walker subunit must trigger substrate binding and HbYX exposure (allowing binding to the 20S) in the neighbouring arginine subunit. This of course assumes the R-finger acts ‘locally’—meaning it only affects the conformation of its own subunit (Fig. 7b). Alternatively, the R-finger could mediate ‘global’ conformational changes—meaning it affects the conformation of all, or most, subunits in the ring to elicit these effects. However, mutation of these conserved arginines does not disrupt: (1) ATP-binding affinity, ATP-binding cooperativity or ATP-binding pattern, which requires global allosteric control—since all six subunits are involved (that is, 2—high, 2—low and 2—no affinity subunits at saturation; Figs 3 and 4a,c, and ref. 8). Such global control of ATP binding is also consistent with a recent ClpX study showing that large/small domain contacts between neighbouring subunits controlled a rigid-body motion for global conformational changes of the ClpX ATPase ring^28^,^45^. These results therefore demonstrate that the dual-R-finger function can be decoupled from the global allosteries that regulate ATP-binding kinetics and the ATP-binding pattern. Therefore, the effects are necessarily limited to individual subunits (or between a subunit and its neighbour), which we consider a ‘local’ effect. In contrast, a study of the R-finger in covalently linked ClpB oligomers indicated it was involved in ATP-binding kinetics and was required for the allosteries regulating ATP-binding cooperativity^46^, which we do not observe for PAN. However, the homohexameric AAA+ ATPase NtrC1 crystal structure showed a PAN analogous ‘local’ acting mechanism for its R-finger, whereby the arginine, upon contacting the neighbour-bound ATP, induces a local conformational change in a surface loop that triggers substrate (*σ*54) binding^47^. Our data imply substrate binding to PAN and HbYX–20S association occur in the arginine subunit and not the Walker subunit, as we and others had assumed previously^8^,^41^. Because substrate binding to the arginine subunit is critical for the mechanochemical coupling that drives protein translocation, the organization of ATP-binding and hydrolysis pattern must be critical to understanding the mechanisms of the proteasomal ATPases (see below).

The FRET analyses presented here show that PAN and the 26S ATPases bind ATP at apparently only neighbouring subunits (Fig. 1), and prior experiments showed they both have similar types of high- and low-affinity subunits. Together these results demonstrate that PAN and the 26S ATPases share a similar ‘global’ allosteric system that regulates the nucleotide-binding pattern. In addition, while the distance between the low-affinity sites (ADP sites) could not be empirically determined with FRET, we can logically conclude that once both ortho-bound ATPs are hydrolysed they become ortho-bound ADPs. It is important to note that several recent 26S proteasome cryo-EM structures show its ATPases have a right-handed helical staircase topology (defined by the vertical substrate-translocating pore loops’ position)^21^,^48^, which is complementary with the ‘ortho’ ATP-binding pattern (Fig. 7b). This helical topology is observed in both the apo state with ATP_h_ (refs 21, 48) and in the translocation-competent state (that is, with either substrate^7^ or ATPγS bound^5^). A three-subunit rotation of the helical topology (a conformational transition) is required to convert between these two helical states. Because ATP binding and hydrolysis must drive the conformational changes that produce work, the individual subunit’s conformation around the ring must be tightly linked to their bound nucleotide. It is expected then that helical topology rotation with substrate bound to the proper pore loop(s) would result in substrate translocation through the ATPase ring. Because nucleotide-binding pattern and helical topology appear to be regulated globally (and mirror one another to some extent), we postulate that the helical topology regulates the nucleotide-binding configuration. So how might the ‘ortho’ binding pattern best fit the topology of the known helical states? Since unidirectional translocation (into the 20S) requires that substrates bind near the top of the ATPase ring (N-terminal side) then be moved downwards into the pore upon ATP hydrolysis^41^, we expect that the two highest positioned subunits are ATP bound, since substrate binding requires the ATP-bound state. On the basis of this, we place the ATP-bound subunits at the top of the lockwasher topology in our working model (Fig. 7c). In addition, ATP hydrolysis to ADP must generate work on the substrate by translocating it^10^,^49^, and thus subunits with pore loops vertically lower in the ring should be the ADP-bound subunits. For model-building purposes, we place the pair of ADP subunits clockwise to the ATP subunits rather than counterclockwise (Fig. 7b,c), and it will become evident below that this arrangement combined with our new understanding of the arginine mediator allows the most coherent model for translocation.

There are two possible mechanisms for ATP hydrolysis progression with an ortho ATP-binding pattern: (1) whereby concerted subunit pairs hydrolyse ATP and sequentially progress around the ring (paired progression), or (2) where ATP binds two subunits, but only one hydrolyses its ATP at a time with sequential progression around the ring (single-subunit progression). Since only ATP can stimulate substrate binding of GFP–ssrA or FITC-casein^8^, at least one bound ATP is required to maintain PAN in the substrate-bound state. Therefore, if PAN hydrolysed both of its ATPs to ADPs in a paired progression, it will lose its affinity for the substrate, which would allow the substrate to slip out of the pore, especially since diffusion is quite fast compared with the ATP hydrolysis rates (∼1 s^−1^). In contrast, single-subunit progression would allow at least one subunit to be in the ATP-bound state at all times, implying that the substrate remains bound to the pore loop during ATP hydrolysis. Thus, single-subunit progression supports a far more plausible and efficient mechanism to power protein unfolding. So would it matter which of the two ‘ortho’ bound ATPs hydrolyse first? The only way the ‘ortho’ binding pattern can be maintained throughout a firing cycle (which is most consistent with our FRET measurements) is if the lagging (clockwise) ATP is hydrolysed first (Fig. 7d). This is the simplest model for conformational progression of ‘ortho’ bound subunits and is also allosterically favoured since our data show a positive cooperativity between the ‘ortho’ ATP subunits allowing for a forward-moving chain reaction of ATP binding and hydrolysis that propagates around the ring. The directionality for subunit progression we have built into our working model is based on the counterclockwise R-finger directionality, as it is an important mediator of mechanochemical coupling in PAN. Because the R-finger is required for substrate binding, ATP binding to the Walker subunit must trigger substrate binding in its clockwise arginine subunit (see Fig. 7b). For efficient translocation to occur, the substrate-bound subunit must maintain its substrate-bound state in both the ATP- and ADP-bound configurations. This would allow the subunit to hydrolyse ATP to ADP and to do work on the substrate by moving it downwards. This aspect is similar to the proposed model of the m-AAA protease’s requirement for maintaining ATP binding for substrate gripping while processing a substrate^50^. Only hydrolysis in the lagging ATP-bound subunit would allow for maintenance of the substrate-bound state during ATP hydrolysis, since its R-finger remains engaged with ATP in the arginine subunit (Fig. 7b,d). On the basis of this logic, the two ADP subunits must be clockwise neighbours from the ATP-bound subunits. Thus, in this model, ATP binds the lagging empty subunits after the lagging ADP leaves (because two subunits are always empty), progressing the helical topology by a single subunit and translocating the substrate by a single step. So, ATP hydrolysis progression around the ATPase ring, mirroring the rotating helical topology, would be counterclockwise (Fig. 7c,d).

Our findings demonstrating the *trans*-acting nature of the R-finger as well as the ortho ATP-binding configuration illuminate an important and perhaps underappreciated necessity for efficient protein translocation through a pore. To visualize lockwasher topology progression and how this relates to the arginine and Walker subunits’ function, we have generated a translocation cartoon model (Fig. 7e) based on the culmination of these findings and discussion. This model incorporates the ‘ortho’ ATP-binding pattern and helical topology with the R-finger along with single-subunit progression to depict how these elements combine to generate a surprisingly simple working model for protein translocation. The hydrolysing arginine subunit (Fig. 7e, subunit C near the top) can only maintain ‘grip’ on substrate if the Walker subunit (subunit B, top) remains in the ATP-bound state. Only the single-subunit progression of the ‘ortho’ ATP-binding pattern, combined with the ‘*trans*’ allosteric effects of the R-finger on substrate binding, can support this type of mechanism. We imagine ATP hydrolysis (that is, conversion to the ADP-bound state) promotes global conformational rotation of the helical topology by one subunit pulling the substrate downwards. Then, subunit-D releases substrate after ATP hydrolysis since its R-finger is no longer engaged with ATP. Cooperative ATP binding to the empty subunit-A would restart the cycle and keep the helical topology moving in a counterclockwise direction. This proposed model for protein translocation and the associated ATP-binding/exchange model are consistent with the function of a local acting R-finger functioning ‘*in trans*’, the ‘ortho’ nucleotide-binding pattern, the observed positive cooperativity and the lockwasher-like conformational arrangements seen in the 26S ATPases.

Cellular ATP concentration is greater than 10 times the affinity for the low-affinity ATP-binding sites, implying it is possible for PAN (or the 26S) to exist in a four-bound state^8^. However, our data suggest PAN normally only binds two ATPs during hydrolysis. What evidence supports this? (1) The Hill coefficient for ATP is approaching 2 (*h*=1.7±0.07), (2) the presence of 2—high, 2—low and 2—no affinity subunits, (3) the saturated four-bound ATP-state functions sub-optimally compared with the two-bound state (Supplementary Fig. 3a) and (4) ADP leaving is thought to be rate limiting. The fourth point is important because PAN cannot bind more than four nucleotides. Therefore, even at cellular saturating conditions, for a new ATP to bind it must first wait for ADP to leave. This implies PAN normally hydrolyses ATP while ADP is bound and thus a four-bound ATP state would not exist even at saturating ATP. In addition, if a four-bound state exists, we would predict four of the six pore loops bind the substrate simultaneously. If this were the case, rapid hydrolysis in all four subunits would only result in translocation by a single step (for example, in the game of tug of war, when four people on one side of the rope take one step back, the rope only translates by one step). Such a mechanism seems inefficient, and does not agree with single-molecule force experiments of ClpX showing it can take 1–4-nm translocation steps, which are interpreted as 1–4-subunit ATP hydrolysis bursts from a four-ATP-bound state^44^,^51^. An alternative interpretation is to assume that new ATP occasionally binds during the step burst (especially at saturating ATP), allowing for longer than 2-nm steps, while never binding more than two ATPs at a time, which is expected for single-subunit progression. However, PAN and ClpX are quite different AAA ATPases (for example, ClpX has a *cis*-functioning sensor 2 arginine, which mediates substrate and ClpP binding, instead of a *trans*-functioning R-finger), and no single-molecule data are yet available for PAN or the 26S ATPases, so further studies are needed to make these comparisons.

Most AAA+ ATPases show a high level of coordination between subunits, and taking into account the strict allosteric constraints of nucleotide binding, we predict that the proteasomal ATPases function by an ordered sequential mechanism. However, studies of the 26S proteasome^15^,^41^ and ClpX^28^,^29^,^44^,^51^ with various combinations of deactivated subunits suggested that subunit skipping can occur. A partial probabilistic ATP hydrolysis model with a degree of intersubunit coordination was developed for ClpX to explain such results^28^,^29^,^44^,^51^. However, our FRET measurements indicate ATP only binds neighbouring subunits, inconsistent with a probabilistic model. How do we rectify these observations with the data presented here? It is well understood that ligand binding is a thermodynamic equilibrium process, and we have shown here that ATP off-rates (2.6 s) are similar to ADP off-rates (3.7 s), which are comparable to the catalytic rates (∼1 ATP per s per hexamer; Fig. 6c). Thus, any one subunit in the hexamer hydrolyses ATP every 6 s (on average), but after binding ATP it leaves after 2.6 s (on average) even when it is not hydrolysed. So, when a mutated subunit is reached in a sequential cycle only seconds, on average, have to pass before ATP leaves to allow new ATP to bind a new functional site, thus resetting the ring and allowing for continuation of the sequential cycle. In this sense, ring resetting can be expected simply based on the thermodynamics of nucleotide binding, and thus its relevance may only be evident when mutations are introduced that impair normal function. While the extent of such thermodynamic ring resetting is unknown during normal operation, if such resetting occurs frequently in a WT enzyme, we expect a probabilistic model would be needed to describe function. However, if it is a rare event, then a sequential model is sufficient to describe the ATPase ring’s inherent operations. In other words, we hypothesize that these ATPases function sequentially between thermodynamic ring-resetting events whenever they might occur. On the basis of these data and rational, it is our model that the proteasomal ATPases function by a sequential mechanism generated by the inherent global allosteries of the multisubunit complex, but similar to all enzymes its mechanism is subject to standard thermodynamic considerations.

## Methods

### Materials and protein purification

PAN, GFP–ssrA, T20S and LFP were prepared as described^10^,^17^. Expression vectors for the PAN arginine mutants (R328A, R331A and R328/331A) in pRSETA were generated by site-directed mutagenesis and were confirmed by sequencing. The purest available forms of ATP, ATPγS and ADP were purchased from Sigma and were stored at −80 °C until use. Mant***-***ATPγS and Mant-ADP were purchased from Jena Bioscience. Mant-ATP and TNP-ATP were purchased from Molecular Probes. Bovine 26S proteasome was purified by the previously described UBL-UIM method^52^ and were exchanged with reaction buffer by rapid spin column or by dialysis (4 h) immediately before use.

### 20S assays

Enzymatic reactions with archaeal proteasomes and ATPase complexes were performed at 45 °C. To measure 20S gate opening, the internally quenched fluorogenic peptide substrate (LFP) was dissolved in dimethylsulphoxide and used at a final concentration of 10 μM in the presence of the indicated nucleotide (ATPγS). GFP–ssrA substrate unfolding and degradation was monitored by GFP emission changes at 509 nm.

### Steady-state nucleotide-binding affinity

Mant***-***ATPγS (and other labelled nucleotides) binding to PAN were analysed as described previously with slight modifications^8^. Briefly, mant-ATPγS binding to PAN was monitored by increase of fluorescence by protein binding at Ex 360 nm/Em 440 nm on a BioTek synergy mx 96-well plate reader. The reaction was run at room temperature in 50 mM Tris (pH 7.5), 5% glycerol and 20 mM MgCl_2_ with the indicated concentration of PAN and nucleotide (0.015 μM).

### Substrate binding

Substrate binding to PAN was monitored by measuring FP. Each WT and mutants PAN protein (0.1 μM) was added to GFP–ssrA (0.08 μM) in the presence of 1 mM ADP or 1 mM ATPγS in 50 mM Tris (pH 7.5), 10 mM MgCl_2_ and 1 mM dithiothreitol. After 20 min incubation at 25 °C, FP was measured in 96-well plates in a Synergy2 BioTek plate reader (Ex=494 nm/Em=515 nm). The anisotropy equation used by the biotech software is as follows: *r*=(Ivv−Ivh)/(Ivv+2Ivh).

### ATPase assays

PAN’s ATPase activity was measured by a NADH-coupled ATP regeneration assay system in 50 mM Tris-HCl (pH 7.5), 5 mM MgCl_2_, 10 μg bovine serum albumin, 5% glycerol, 2U/reaction lactate dehydrogenase and 2U/reaction pyruvate kinase (Sigma), 3 mM phosphoenolpyruvate, 0.2 mg ml^−1^ NADH and 2 mM ATP. PAN concentration was 100 nM unless specifically mentioned in the legend. ATPase activity was followed by a loss of NADH absorbance at 340 nm. Kinetic analysis of ATP hydrolysis was done by varying the ATP concentrations (10–4 mM) with a fixed concentration of PAN (100 nM). *V*_max_, *K*_M_ and Hill coefficient (*h*) values were obtained by nonlinear regression analysis using the Hill equation (Sigma plot).

### Nucleotide-binding stoichiometry

Stoichiometry of ATPγS binding to the indicated amount of PAN was determined as described previously with modifications using fluorescent nucleotide (mant-ATPγS) instead of a radiolabelled ATPγS^8^. Briefly, different concentrations of mant-ATPγS were incubated with PAN at room temperature, and the bound complex was rapidly separated (in approximately <1 s) from the free nucleotide by 100 μl Sephadex G50 spin column. In total, 2 mM ADP was added to the flow-through to compete off the mant-ATPγS from PAN, so that quantification of the unbound form would be comparable to the independent standard curve, which was used to determine the number of nucleotides bound to the PAN.

### Mant–nucleotide dissociation kinetics

The kinetics of mant***-***ADP and mant-ATPγS dissociation from PAN was obtained by Horiba Fluorolog 3 spectrofluorometer with pneumatically driven SFA-20/SPEX stopped-flow accessories for rapid kinetic acquisitions. In total, 150 nM PAN and 150 nM m-ADP (or mant-ATPγS) were mixed to form a prebound mant–nucleotide complex, which was competed off with excess amount of ADP (2–4 mM) using stopped-flow injection. The dissociation curve of m–nucleotide from PAN was analysed with Oracle by fitting to either single- or double-exponential decay models to derive the dissociation half-life (*T*_1/2_).

### FRET measurements

FRET experiments were done by mixing the 1 μM donor Mant-ATP and 1 μM acceptor TNP (trinitrophenyl)-ATP to the 1 μM PAN in assay buffer (50 mM Tris-HCl (pH 7.5), 10 mM MgCl_2_ and 1 mM dithiothreitol) for 20 min and measurements were made by monitoring donor (Mant) fluorescence decrease (exciting at 350 nm and scanning emission spectra between 370 and 650 nm) to observe fluorescence resonance energy transfer on a BioTek 96-well plate reader. This FRET pair was used previously to determine the distance between two bound nucleotides in a G-protein dimer^37^. The acceptor (TNP) fluorescence increase due to FRET was not displayed significantly in emission spectra due to the low quantum yield of TNP fluorescence. All measurements were performed in triplicate, were highly repeatable and the FRET efficiency was calculated by the following equation: *E*=1−*F*_DA_/*F*_D_, where *E* is the FRET efficiency, *F*_DA_ is the fluorescence intensity of the donor with the acceptor present and *F*_D_ is the fluorescence intensity of the donor without the acceptor. In addition, due to the mixing of FRET pairs with equal affinities, *E* had to be corrected by a factor of 0.5 (*E*_corrected_=*E*(1/0.5)), as explained in detail previously^37^. In brief, equal mixing of Mant (M) and TNP (T) nucleotides, would be expected to generate four different types of complexes: two that FRET (MT and TM), and two that do not (MM and TT). In this way 50% of the fluorescent intensity observed comes from the FRET condition and 50% does not, thus requiring this correction factor.

Distance estimates were calculated using the equation, *E*_corrected_=1/{1+(*r*/*R*_0_)^6^}, where *E*_corrected_ is the FRET efficiency calculated above, *r* is the actual distance and *R*_0_ is the Förster critical distance for the FRET pair. For the Mant and TNP pair, *R*_0_ is known to be 40 Å (ref. 37). The *κ*^2^ limits were determined by determining the steady-state (0.17 mant, and 0.25 TNP) and fundamental (0.33 for Mant and TNP) anisotropies of the bound donor and acceptor on a Horiba Fluorolog 3 as described^53^. The calculated *κ*^2^_min_ and *κ*^2^_max_ values are given in Table 1.

## Additional information

**How to cite this article:** Kim, Y.-C. *et al*. ATP binding to neighbouring subunits and intersubunit allosteric coupling underlie proteasomal ATPase function. *Nat. Commun.* 6:8520 doi: 10.1038/ncomms9520 (2015).

## Supplementary Material

Supplementary Information Supplementary Figures 1-5

## Figures and Tables

**Table 1:** Förster resonance energy transfer between high-affinity nucleotides bound to PAN or the 26S proteasome (from Fig. 1).

**Experimental condition**	**FRET efficiency,** ***E***	**D–A distance (Å)** ***r***(***κ***^**2**^**=2/3)**	**D–A distance (Å)** ***r***_**min**_**−*****r***_**max**_(***κ***^**2**^limits)^*^
PAN-E271Q (1 μM) with m-ATP and t-ATP	0.67±0.07	37±2.0	28–47
PAN WT (1 μM) with mATPyS and t-ATP	0.75±0.04	34±1.0	27–44
26S proteasome-WT (1 μM) with mATPyS and t-ATP	0.77±0.08	31±2.6	26–44
PAN-E271Q (1 μM)+GFP–ssrA (1 μM)	0.71±0.09	36±2.8	28–46
PAN-R328/331A (1 μM) with m-ATP and t-ATP	0.65±0.03	37±0.8	29–48

FRET, Förster resonance energy transfer; m-ATP, mant-adenosine triphosphate (donor); t-ATP, TNP-adenosine triphosphate acceptor; WT, wild type.

^*^ϰ^2^ limits were determined by determining the steady-state and fundamental anisotropies of the bound donor (D) and acceptor (A). For example, for PAN-E271Q: *ϰ*^2^_min_=0.14; *ϰ*^2^_max_=2.9.
