# Supplementary material for: ATP binding to neighbouring subunits and intersubunit allosteric coupling underlie proteasomal ATPase function
Source: Nat Commun. 2015 Oct 14;6:8520. doi: 10.1038/ncomms9520 (PMC4608255; doi:10.1038/ncomms9520)
Supplement: Supplementary Information — Supplementary Figures 1-5 [file ncomms9520-s1.pdf]

Supplementary Figure 1

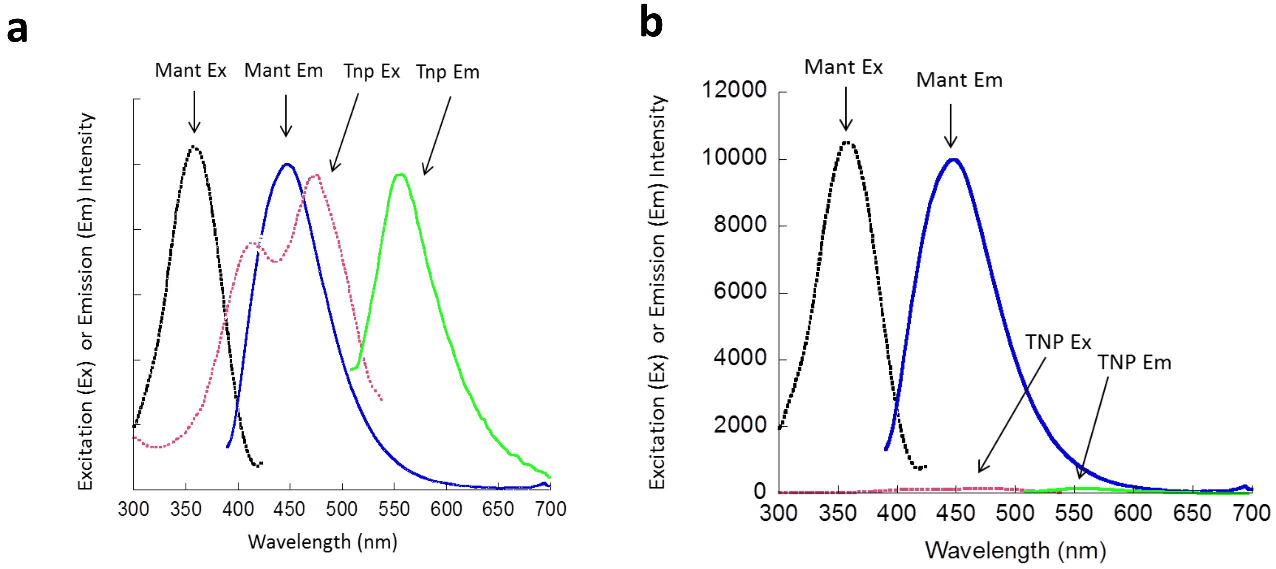

Supplementary Figure 1. Spectra of FRET pair. Normalized (a) and raw (b) absorption and fluorescence spectra of donor (m-ATP) and acceptor (t-ATP) pair used in FRET experiments (Figure 1).

Supplementary Figure 2

a

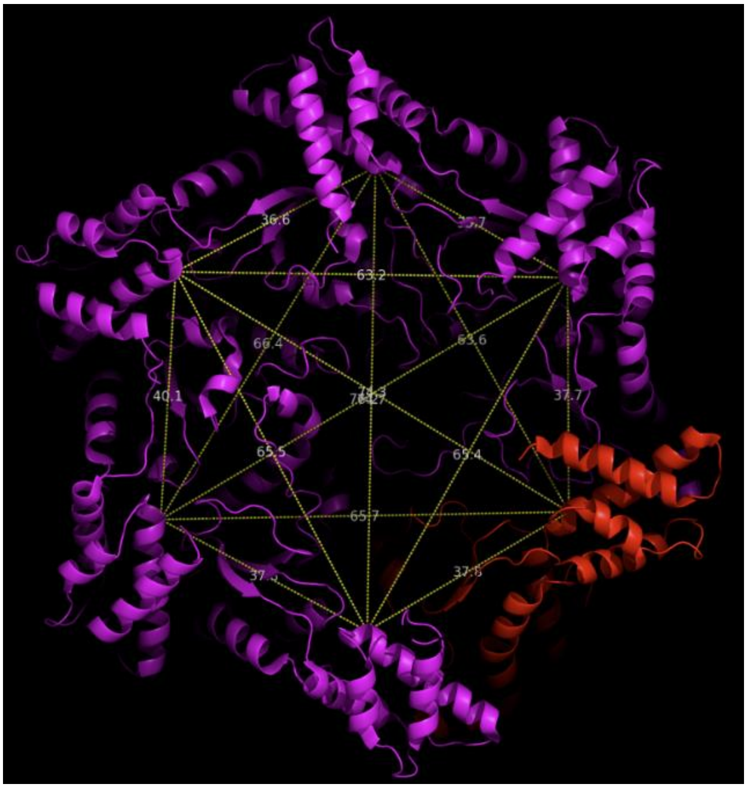

b

Rpt Pair	**Ortho**	Rpt Pair	**Meta**	Rpt Pair	**Para**
2-6	35.7	1-6	63.6	1-3	74.3
1-2	37.7	2-3	63.2	2-4	76.2
5-1	37.8	6-4	66.4	6-5	74.7
4-5	37.8	3-5	65.5		
3-4	40.1	4-1	65.7		
6-3	36.6	5-2	65.4		
Average	**37.6**		**65.0**		**75.1**

Supplementary Figure 2. Structural model of the 26S ATPases and estimated distances between nucleotide binding sites. (a) Structure of the 26S ATPase complex (PDB:4CR4). Distances between the alanine residues in putative Sensor 2 motif were determined using Pymol. The sensor 2 alanine residue was chosen for distance measurements because it contacts the ribose ring of the nucleotide which is the moiety that is modified by the mant or TNP fluorophores and thus best approximates the distance between the bound FRET pair. The ATPase ring is shown with C-terminal side up, and the red subunit is Rpt1. (b) All distances between nucleotide binding sites in the 26S ATPases (PDB:4CR4). Distance is shown in Angstroms and is an estimate since the original source data for the map was determined by cryo-EM to a resolution of ~8Å.

Supplementary Figure 3

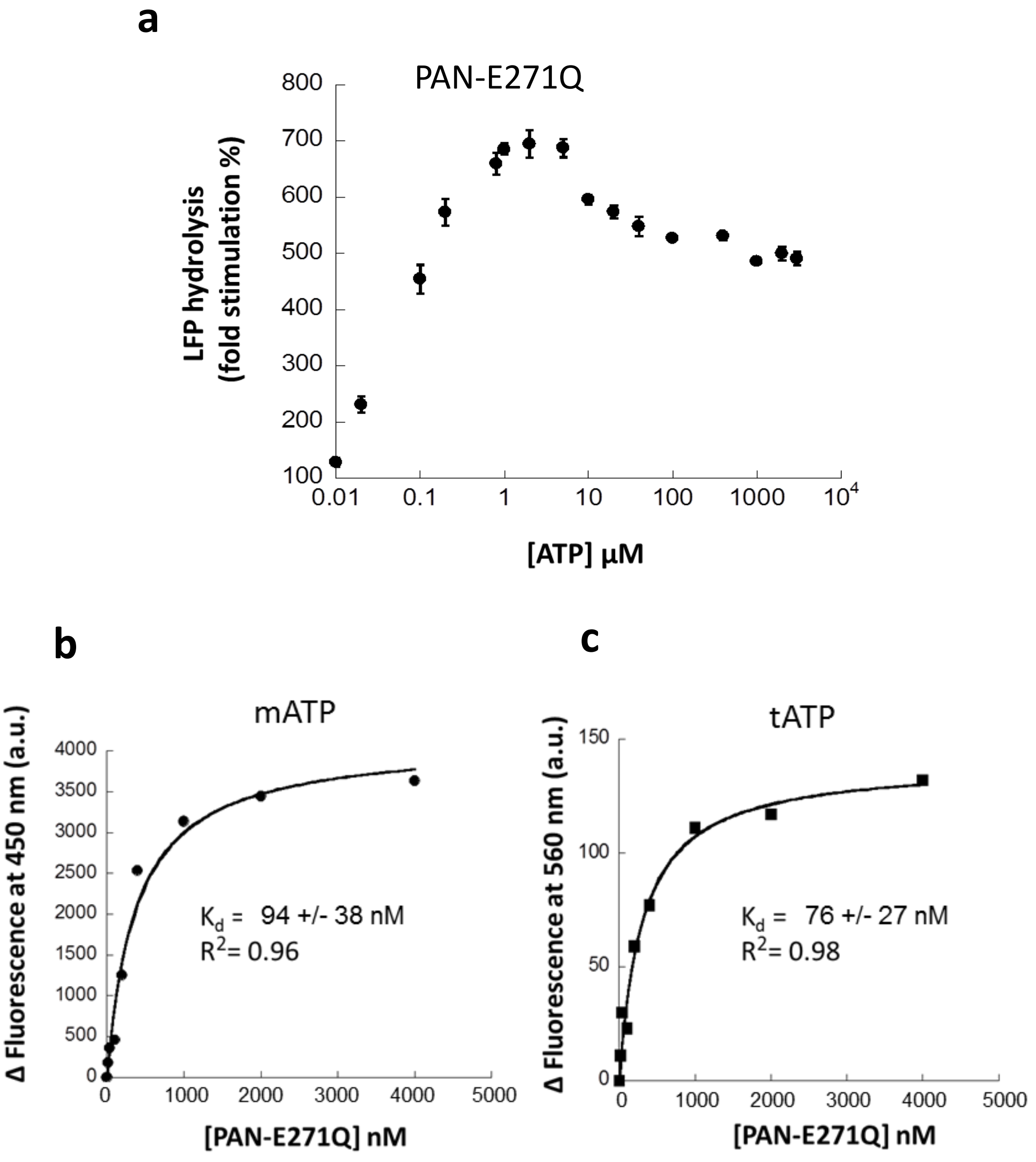

Supplementary Figure 3. Binding of ATP or fluorescently labeled ATP to hydrolysis mutant PAN-E271Q (fluorescent labeling doesn't change affinity to PAN). (a) Gate opening in the 20S proteasome (20 nM) by PAN Walker B mutant (PAN-E271Q) (80 nM) was monitored with the LFP hydrolysis in the presence of a different concentration of ATP. This biphasic curve for 20S gate opening was also observed for ATPγS binding to PAN and correlates with the 2-bound (optimal) and 4-bound (suboptimal) states of PAN (Smith et. al. 2011). The data are the means of three independent measurements ± S.D. (b) Equilibrium ATP binding affinity was determined (for the 2-bound state only) by monitoring the change in fluorescence intensity of mant-ATP (0.05 µM) in the presence of increasing amounts of PAN-E271Q. The 1:1 binding equation (hyperbola) was fit to the raw data points to obtain the shown Kd value. (c) Equilibrium ATP binding affinity was also determined by monitoring the change in fluorescence intensity of TNP-ATP (0.05 µM) by PAN-E271Q binding to obtain Kd value. These Kd values are similar to prior reports for ATP or ATPγS affinity to PAN (Smith et al. 20011).

Supplementary Figure 4

a

```
PAN    314 GATNRPDILDPAILRPGRFDRIIEVP-APD 342
yRpt1  353 FATNRPNTLDPALLRPGRIDRKVEFS-LPD 381
yRpt2  326 MATNKIETLDPALIRPGRIDRKILFE-NPD 354
yRpt3  317 MATNRADTLDPALLRPGRLDRKIEFPSLRD 345
yRpt4  325 MATNRPDTLDPALLRPGRLDRKVEIP-LPN 353
yRpt5  325 AATNRVDVLDPALLRSGRLDRKIEFP-LPS 353
yRpt6  292 MATNRLDILDPALLRPGRIDRKIEFP-PPS 320
hRpt1  319 MATNRPDTLDPALMRPGRLDRKIEFS-LPD 347
hRpt2  329 MATNRIETLDPALIRPGRIDRKIEFP-LPD 357
hRpt3  309 MATNRADTLDPALLRPGRLDRKIEFP-LPD 337
hRpt4  291 MATNRPDTLDPALLRPGRLDRKIHID-LPN 319
hRpt5  332 AATNRVDILDPALLRSGRLDRKIEFP-MPN 358
hRpt6  293 MATNRIDILDSALLRPGRIDRKIEFP-PPN 321
```

b

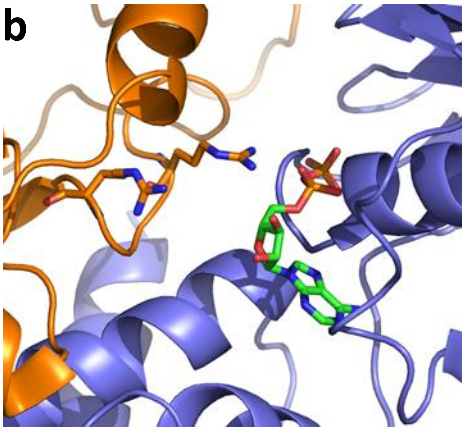

c

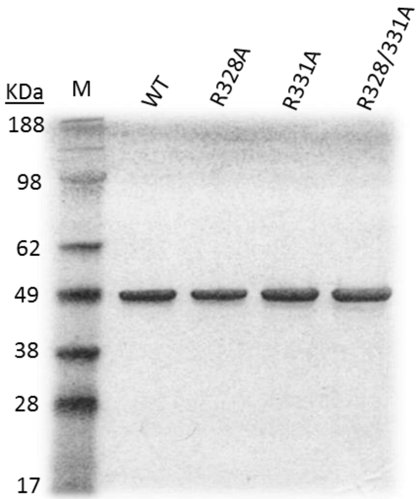

d

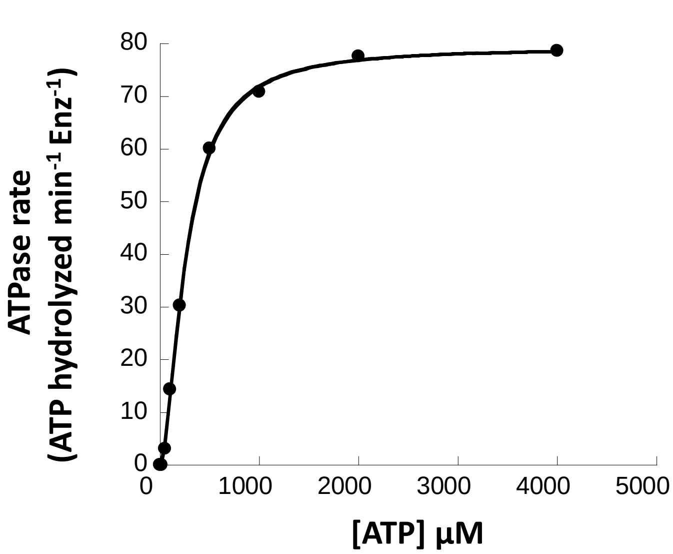

Kinetic constants	V_{max} (min^{-1})	K_M (µM)	h
WT PAN	79.4 ± 0.9	261 ± 8	1.70 ± 0.07

Supplementary Figure 4. Identification of the conserved arginines in the proteasomal ATPases and generation of arginine mutants in PAN. (a) Alignment of the Secondary Region of Homology (SRH) in the proteasomal ATPases from *M. jannaschii* (PAN), *S. cerevisiae* (yRpt1-6), and *H. sapiens* (hRpt1-6), and the conservation of putative arginine finger residues (red and bold). (b) Structure of the Rpt2-1 interface (Rpt2-orange and Rpt1-blue) showing the two highlighted conserved arginines (orange rods; PDB:4BGR). To add a nucleotide to the ATP binding site the crystal structure of PAN with nucleotide was fit to RPT1 (green rods). (c) Purified PAN (WT) and indicated PAN arginine mutants were resolved by SDS-PAGE and stained with Coomassie. The molecular weight marker (M) is shown. (d) The rate of ATP hydrolysis was determined at increasing concentrations of ATP for the WT PAN. The Hill equation was used to fit the data and derive the kinetic constants. Representative data is presented from three independent experiments.

Supplementary Figure 5

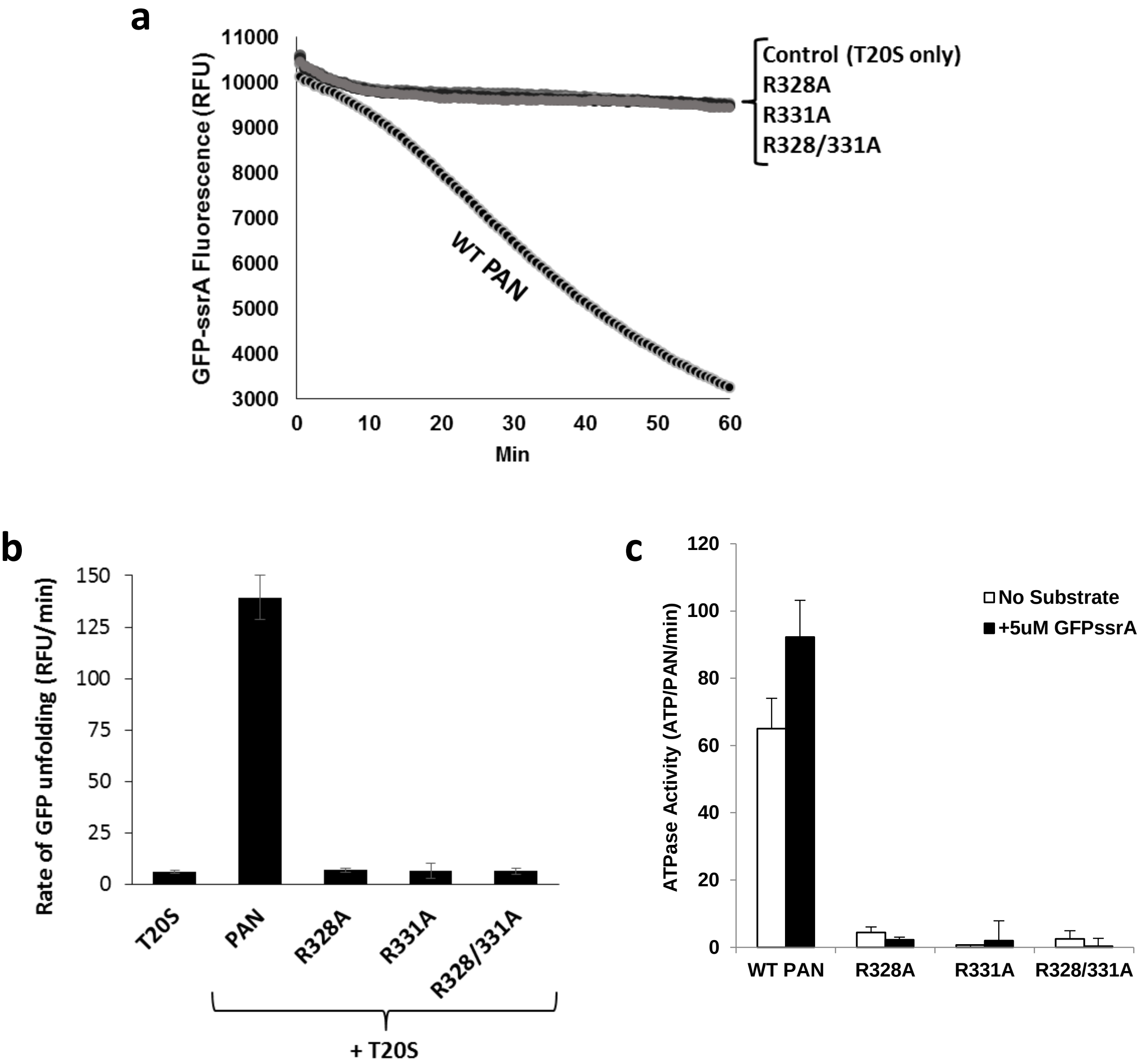

Supplementary Figure 5. PAN's conserved arginines are required for substrate unfolding and substrate stimulated ATPase activity. (a) GFP-ssrA (500 nM) fluorescence (an indication of its native folded state) was monitored in the absence and presence of PAN and the indicated variants (100 nM). All samples contained the T20S proteasome which degrades the unfolded GFP-ssrA preventing its refolding, and is used as a negative control. This shows representative raw data showing the fluorescence intensity over time. (b) The rate of fluorescence decrease (GFP-ssrA unfolding) was determined for each sample in a. (c) the indicated PAN variant was incubated with or without GFP-ssrA and the ATPase activity was monitored. Values are means of three independent measurements +/-S.D.
